# Activation of Peptidylarginine Deiminase in the Salivary Glands of *Balb/c* Mice Drives the Citrullination of Ro and La Ribonucleoproteins

**DOI:** 10.1155/2017/8959687

**Published:** 2017-11-26

**Authors:** Mayra Rodríguez-Rodríguez, Rafael Herrera-Esparza, Juan-José Bollain y Goytia, María-Elena Pérez-Pérez, Deyanira Pacheco-Tovar, Jessica Murillo-Vázquez, Guadalupe Pacheco-Tovar, Esperanza Avalos-Díaz

**Affiliations:** ^1^Department of Immunology, Unidad Académica de Ciencias Biológicas, Universidad Autónoma de Zacatecas, Guadalupe, ZAC, Mexico; ^2^Pharmacology PhD Program, Centro Universitario de Ciencias de la Salud (CUCS), Universidad de Guadalajara, Guadalajara, JAL, Mexico

## Abstract

The goal of the present study was to determine whether peptidylarginine deiminase PAD2 and PAD4 enzymes are present in *Balb/c* mouse salivary glands and whether they are able to citrullinate Ro and La ribonucleoproteins. Salivary glands from *Balb/c* mice were cultured in DMEM and supplemented with one of the following stimulants: ATP, LPS, TNF, IFN*γ*, or IL-6. A control group without stimulant was also evaluated. PAD2, PAD4, citrullinated peptides, Ro60, and La were detected by immunohistochemistry and double immunofluorescence. PAD2 and PAD4 mRNAs and protein expression were detected by qPCR and Western blot analysis. PAD activity was assessed using an antigen capture enzyme-linked immunosorbent assay. LPS, ATP, and TNF triggered PAD2 and PAD4 expression; in contrast, no expression was detected in the control group (*p* < 0.001). PAD transcription slightly increased in response to stimulation. Additionally, PAD2/4 activity modified the arginine residues of a reporter protein (fibrinogen) *in vitro*. PADs citrullinated Ro60 and La ribonucleoproteins *in vivo*. Molecular stimulants induced apoptosis in ductal cells and the externalization of Ro60 and La ribonucleoproteins onto apoptotic membranes. PAD enzymes citrullinate Ro and La ribonucleoproteins, and this experimental approach may facilitate our understanding of the role of posttranslational modifications in the pathophysiology of Sjögren's syndrome.

## 1. Introduction

In certain autoimmune diseases, components of the immune response recognize epitopes at peptidylarginine to peptidylcitrulline posttranslationally modified residues. This catalytic pathway involves the activity of peptidylarginine deiminases (PADs), which induce a hydrolytic reaction in arginine that results in protein citrullination. This stereochemical process modifies the physicochemical behaviour of the target protein by increasing its antigenicity [1].

PADs are a family of calcium-dependent enzymes that possess the following isoforms: PAD1, PAD2, PAD3, PAD4, and PAD6, which are encoded by four different genes [2]. PADs are involved in basic processes of cell physiology such as fertilization and embryo development; the citrullination of histones by PAD4 antagonize the histone methylation induced by the histone arginine methyltransferase and affect the gene expression [3–5]. Likewise, PADs participate in the terminal differentiation of the skin [6].

Deregulation of PAD activity is involved in the pathophysiology of autoimmune diseases, and protein citrullination is considered an extraordinary mechanism for producing autoantigens of clinical importance in rheumatoid arthritis, optic myelitis, and other autoimmune diseases. Additionally, the citrullination of proteins and histones plays a role in the pathogenesis of cancer [7].

Sjögren's syndrome is an autoimmune disease that primarily affects the salivary and lachrymal glands. Clinically, it manifests as dryness of the eyes and mouth. The pathogenic immune mechanisms underlying Sjögren's syndrome initially involve the innate immune system. Subsequently, adaptive immune mechanisms activate B and T cells, with the production of B cell activating factor, which is a member of the TNF family. Together with interferon (IFN*γ*), B cell activating factor contributes to cellular infiltration, which induces the formation of lymphocytic foci in the salivary glands the hallmark of Sjögren's syndrome [8].

In rheumatoid arthritis (RA), citrullinated peptides trigger an autoimmune reaction that results in the production of cyclic citrullinated peptide antibodies (CCP antibodies), which is considered to be an early specific marker of RA. In primary Sjögren's syndrome, 10% of patients are positive for CCP antibodies [9]. Nevertheless, the origin and pathological significance of CCP antibodies in Sjögren's syndrome are merely speculative. Therefore, we studied the salivary glands of patients with Sjögren's syndrome to identify the catalytic machinery responsible for protein citrullination. Our previous findings revealed the presence of PAD enzymes and citrullinated proteins in the salivary glands of patients with Sjögren's syndrome [10].

Given the aforementioned results, we hypothesized that under certain stress conditions, PAD enzymes in the salivary glands might be activated and citrullinated Ro60 and La ribonucleoproteins, which are considered to be relevant antigens in Sjögren's syndrome. The Ro60 protein is donut-shaped that displays binding sites for RNA, this ribonucleoprotein has localization in the nucleus and cytoplasm, and its function depends on where it is located. Ro60 ribonucleoprotein is associated with one or more hYRNAs; therefore, these small noncoding RNAs may form complexes with Ro60 and probably they have the function of cavity keeper Ro60 in preventing binding of misfolded ncRNAs [11]. La or SSB autoantigen is a 48 kDa protein that works as a transcription termination factor of RNA polymerase III; therefore, La ribonucleoprotein participates in the folding and maturation of RNA polymerase III transcripts, protecting them against the exonuclease degradation, and this ribonucleoprotein is critical to stabilize some nascent pre-miRNA [12]. Hence, anti-Ro antibodies in Sjögren disease can be detected in 70–100% of patients, while La/SSB in the 40–90%, respectively [13]; in consequence, it is of interest to define whether PAD enzymes could affect the expression and antigenic modification of these ribonucleoproteins which are major targets of the autoimmune response in Sjögren.

However, it was difficult to test our hypothesis because the *in vivo* conditions required for the activation of PAD enzymes are unknown. Furthermore, it is equally difficult to obtain salivary glands from patients with Sjögren's syndrome to develop *in vitro* assays.

Because different molecules, such as ATP, LPS, and TNF, can activate PAD enzymes *in vitro* and such molecules may simultaneously activate the inflammasome, a pathway capable of inducing apoptosis [14–16], we designed a model of PAD enzyme activation using the parotid glands of female *Balb/c* mice and different molecules to stimulate PAD activity, this model is simple, because these animals possess big parotid glands of easy access for surgical extraction.

The aim of the present study was to answer the following questions: (1) Are PAD2 and PAD4 enzymes present in mouse salivary glands? (2) Is it possible to induce PAD activity? (3) If so, is PAD activity dependent on transcription? (4) Are the induced PAD enzymes functional? (5) Are the induced PADs able to citrullinate Ro and La ribonucleoproteins?

To address these questions, the salivary glands of *Balb/c* mice were cultured and stimulated with different molecules, and PAD enzymes and citrullinated proteins were assessed.

## 2. Materials and Methods

### 2.1. Tissue Culture

The parotid glands from five female *Balb/c* mice were obtained by dissection and were used in each experimental condition. The animals were euthanized, and then the parotid glands were excised under aseptic conditions. The mass of tissue was standardized by weight, and also after extraction, protein and mRNA were standardized by spectrophotometry in each salivary gland sample. All experimental procedures with animals were performed according to the Mexican Guidelines for the Production, Care, and Use of Laboratory Animals (NOM-062-ZOO-1999) and the International Guide for the Care and Use of Laboratory Animals, and experiments were performed according to the guidelines for ethical conduct in the care and use of animals developed by the American Psychological Association (APA) (http://www.apa.org/science/anguide.html). The protocol number UAZ-2013-36474 for animal experiments was approved by the Bioethics Committee of our Institution.

The tissues were rinsed with sterile phosphate-buffered saline (PBS) and cultured in Dulbecco's modified Eagle's medium (DMEM) (Sigma, St. Louis, MO) for 3 hours at 37°C in a 5% CO_2_ atmosphere. The culture medium was supplemented individually with the following stimulant molecules by group (*n* = 5): (A) 2 mM ATP, (B) 2 *μ*g/mL of LPS, (C) 200 ng of TNF, (D) 250 UI of IFN*γ*, (E) 200 ng of IL-6, and (F) in basal conditions without stimulant molecules; aforementioned concentrations of the stimulant molecules were defined in basis of previous reports by other investigators (14–16). After the incubation, the tissues were processed as follows: one parotid gland was immersed in Tissue-Tek®, frozen at −20°C, and the cryosections were used for direct immunofluorescence. Another parotid gland was fixed in 10% formalin and processed for H&E staining and immunohistochemistry. Another set of parotid glands was used for protein and RNA extraction, as well as for PAD isolation for functional enzymatic assays.

### 2.2. CCP Antibodies

ELISA assays were used to detect antibodies against citrullinated synthetic proteins. Serum from a patient with rheumatoid arthritis was used as a positive control, and sera from various patients with primary Sjögren's syndrome were also tested. The assays were performed according to the manufacturer's instructions (Euroimmun AG, Luebeck Germany). Serum samples diluted 1 : 100 were incubated for 60 minutes at room temperature in wells of polystyrene plates coated with synthetic cyclic citrullinated peptides (CCPs) [17]. Next, the plates were washed with washing buffer, and the bound antibodies were incubated with conjugated HRP-rabbit polyclonal anti-human IgG (horseradish peroxidase) for 30 minutes. Finally, the colour reaction was developed by incubating the sample for 30 minutes with the developer pNPP (p-nitro phenyl phosphate, disodium salt). The reaction was stopped, and the plates were assessed using an ELISA reader at 450 nm. In all experiments, five calibrators of the kit were used to construct the standard curve, and the results were expressed in densitometric units. All assays were performed in triplicate.

### 2.3. Purification of High-Affinity CCP Antibodies and Peroxidase Labelling

Serum from a patient with primary Sjögren's syndrome who was positive for CCP antibodies was subjected to ELISA as described previously [18] with the following modifications: after incubation, specific CCP-bound antibodies were eluted from the CCP-coated polystyrene plates with 0.2 M glycine-HCl at a pH of 2.8. The pH was neutralized with 1 M Tris, pH 9.5. This solution of purified high-affinity CCP antibodies was concentrated in an EMD Centriplus centrifugal concentrator with a 30 kDa molecular weight cut-off (Fisher Scientific, USA). The protein concentration was measured using the Bradford method [19].

The purified high-affinity CCP antibodies were labelled with horseradish peroxidase (HRP) (Sigma, St. Louis, MO) using the glutaraldehyde method [20]. The molar ratio of peroxidase to antibodies was 1 : 10. Briefly, one milligram of the enzyme was dissolved in 0.3 M sodium bicarbonate pH 8.1 and was preactivated with 500 *μ*L of 1% glutaraldehyde and incubated with the sample at 37°C for 30 minutes. Excess of glutaraldehyde was quenched with 100 *μ*L 1-fluoro-2,4-dinitrobenzene (Sigma, St. Louis, MO) dissolved in absolute ethanol, and then the mixture was oxidized with 0.08 M sodium periodate to induce the aldehyde groups, such reaction was stopped by the addition of 0.16 M ethylene glycol (Sigma, St. Louis, MO) in distilled water; the mixture was dialyzed in against 0.01 M sodium carbonate buffer. Additionally, protein concentration of CCP antibodies was determined at 280 nm; then, 10 mg of antibodies was coupled to peroxidase aldehyde. The coupling reaction was induced over an 18-hour incubation at room temperature in sodium carbonate buffer pH 9.5, and this reaction was stabilized with 1 mg of sodium borohydride (Sigma, St. Louis, MO). The CCP antibody/HRP conjugate was dialysed extensively against distilled H_2_O using a membrane with a pore size of 40 kDa. The CCP antibody/HRP label was then fractionated in a Sephadex G-200 (Sigma-Aldrich, St. Louis, MO) minicolumn equilibrated with PBS. Fractions of 100 *μ*L were collected, and the protein concentration in each fraction was determined at 280 nm. ELISA was performed to evaluate CCP antibody activity. The affinity-purified HRP-labelled CCP antibodies were concentrated using a Centriplus concentrator and used for immunohistochemistry; the final yield conjugated antibody concentration was of 6 mg. Another fraction of affinity-purified unlabelled CCP antibodies was used for the double immunofluorescence assays.

### 2.4. Immunohistochemistry

PAD2 and PAD4 enzymes, as well as Ro60 and La ribonucleoproteins, were detected in 4 *μ*m thick sections of parotid salivary glands. The tissue specimens were deparaffinized, permeabilized with 0.01% Triton X-100/phosphate-buffered saline, and washed three times with PBS. Endogenous peroxidase was quenched for 10 minutes with 3% H_2_O_2_ in methanol, followed by a blocking step with 3% foetal calf serum (Gibco, Thermo Fisher Scientific). The tissues were washed and incubated for 12 hours with the appropriate dilution of specific anti-PAD2, anti-PAD4, anti-Ro60, and anti-La antibodies. The slides with the tissues were washed extensively with PBS and then incubated for 2 hours with the secondary antibody. The HRP-labelled antibodies were detected by development with 3,3-diaminobenzidine 0.06% H_2_O_2_ (Sigma, St. Louis, MO), and the reaction was stopped with 0.5 M sulfuric acid. The slides were examined by light microscopy. All assays were performed in triplicate and evaluated in a blinded manner. Additionally, citrullinated proteins were detected by incubation for 12 hours with affinity-purified HRP-labelled CCP antibodies diluted 1 : 50. After extensive washing with PBS, the colour reaction was developed as previously described and evaluated by light microscopy in a blinded manner [21].

### 2.5. Antibodies

The following antibodies were used: anti-PAD2 antibody (PA5-19474, Thermo Scientific; diluted 1 : 100) and anti-PAD4 (PA5-2217, Thermo Scientific; diluted 1 : 100), anti-Ro60 (H-300 Sc-20961) or anti-La (sc-166274, Santa Cruz Biotechnology; diluted 1 : 100), and anti-*β*-actin (ab2072 Abcam). Peroxidase-conjugated goat anti-mouse IgG (Sigma, St. Louis, MO) or goat anti-rabbit IgG (Abcam ab759) was used as secondary antibody. Additionally, an anti-citrulline antibody (231246, Calbiochem, Darmstadt, Germany; 1 : 100 dilution in 10% foetal bovine serum (FBS) PBS) was used for immunohistochemistry.

### 2.6. Primer Design

PAD2, PAD4, Ro60, and La gene expression was detected by qPCR amplification from genomic RNA obtained from *Balb/c* salivary glands with the following primers: PAD2 forward 5′-GCC TCG ACT CCT TCG GGA-3′ and reverse 5′-ACG GGG TAC TCC TTG CCA T-3′, PAD4 forward 5′-TGG AAG GTC TTG CTT TCC CA-3′ and reverse 5′-TCC AGC AGG GAG ATG GTG A-3′ [22], Ro60 forward 5′-TCA CAT CTT AAA CCT TCC AGT GA-3′ and reverse 5′-ACTT AAC ATA TTT CTT TTT GTG AGA G-3′, La forward 5′-GAT GAA AAT GGT GCA ACT GG-3′ and reverse 5′-CTG TTT TCT GTT GTT TGG GAT GC-3′ [23], *β*-actin forward 5′TGG AAG GTC TTG CTT TCC CA-3′ and reverse 5′-TCC AGC AGG GAG ATG GTG A-3′ [24], and 18SrRNA forward 5′-CTA CGT CCC TGC CCT TTG TAC A-3′ and reverse 5′-ACA CTT CAC CGG ACC ATT CAA-3′ [25], which was used as a housekeeping gene for qPCR analysis.

### 2.7. Generating cDNA by RT-PCR

Total RNA (250 *μ*g) was isolated with TRIzol*®* Plus RNA purification (cat. 12183-555, Ambion™ by Life technologies) and standardized by UV spectrophotometry at 260 nm; RNA was mixed with 200 *μ*M dNTP, 0.7 *μ*M of the reverse primer, and 5 U/20 *μ*l of rTth/DNA polymerase (SuperScript™ IV reverse transcriptase, Invitrogen, Thermo Fisher Scientific) and incubated at 70°C for 10 minutes. Amplification of the PAD2, PAD4, Ro60, La, *β*-actin, and 18SrRNA cDNA was conducted by PCR with 0.15 mM of the forward primer consisting of 35 cycles of 94°C for 15 sec, 60°C for 30 sec, and 72°C for 1 minute. The PCR products were electrophoresed in a 2.7% agarose gel containing 0.5 *μ*g/ml ethidium bromide, and the gels were analysed using a Molecular Imager ChemiDoc™XRS (Bio-Rad).

### 2.8. Quantitative Real-Time PCR (qPCR)

qPCR was done by incubation of 20 ng of cDNA (standardized by spectrophotometry at 260 nm) with 10 *μ*l of Fast SYBR® Green Master (Thermo Fisher Scientific) mix adjusting the final volume to 20 *μ*l with H_2_O; mixture reaction was loaded by triplicate in a 48 wells micro plate (Mini Plate Spinner, MPS 100, Lab Net), and after 30 sec centrifugation, the reaction was done in a Pixo Helixis system (Illumina San Diego, CA, USA) consisting in an initial PCR activation of 20 sec at 95°C, followed by 35 cycles of denaturation 3 sec at 95°C, annealing 30 sec at 60°C and extension 30 sec at 60°C except for Ro60 and La which was at 52°C. Relative quantification was done by the comparative method (ΔCt). The expression level of each gene was normalized using 18S gen as invariant endogenous control.

### 2.9. Activation of PAD2 and PAD4 Proteins in Salivary Glands

The expression of PAD2 and PAD4 proteins was assessed in salivary gland extracts treated with ATP, LPS, TNF, IL6, or IFN*γ*. Briefly, after culturing, the tissues were washed with sterile cold PBS, pH 7.2. The tissues were disrupted by sonication in 200 *μ*l of lysis buffer (1% Triton X-100, 140 mM NaCl, 1 mM EDTA, 10 mM Tris-HCI, pH 7.6, and 1 mM PMSF). The extracts were then centrifuged at 14,000 rpm for 10 minutes at 4°C, and the supernatants containing soluble fractions of salivary glands were saved and used to detect PADs by ELISA, in functional assays, and by Western blot analysis.

### 2.10. SDS-PAGE and Western Blot Analysis

After protein quantification by spectrophotometer by the Bradford method at 595 nm and by UV coefficient extinction at 280 nm, cell extracts were separated in 10% SDS-PAGE gels. In each experimental condition, tissue extracts were standardized to load 10 *μ*L of a 2 mg/mL final concentration of protein per well [26], after electrophoresis proteins were transferred onto nitrocellulose membranes (Hybond-C, Amersham, UK), and then nonspecific binding of the antibodies was blocked with 5% nonfat dry milk dissolved in PBS [27]. Immunoreactive bands were identified by applying the appropriate dilution of primary antibodies of specific anti-PAD2 or PAD4 and anti-Ro60 (H-300 Sc-20961) or anti-La (sc-166274, Santa Cruz Biotechnology) in 2% nonfat dry milk, 0.3% Tween® 20, PBS, and membranes with primary antibodies that were incubated and rocked gently overnight at 6°C. Peroxidase-conjugated goat IgG or anti-mouse IgG (Sigma, St. Louis, MO) served as the secondary antibody. After a 1-hour incubation, immunoreactive bands were detected using chemiluminescence (ECL, RPN2106; Amersham). Moreover, citrullinated proteins were detected using an anti-citrulline antibody (231246, Calbiochem, Darmstadt, Germany; 1 : 100 dilution in 10% foetal bovine serum (FBS) PBS). Chemiluminescent Western blot detection was used according to the manufacturer recommendations (Immun-Star Western C chemiluminescence kit, Bio-Rad, catalogue 170-5070), the protein band densities were determined in a Molecular Imager ChemiDoc XRS (Bio-Rad), and normalized values were calculated using as control protein *β*-actin dividing all normalizing control with each protein of interest.

### 2.11. Immunoprecipitation Assay

Immunoprecipitation assay was done using an anti-citrulline antibody adsorbed by Sepharose 4B-SPA (Sigma, St. Louis, MO, USA)-yielded immunoprecipitates containing diverse citrullinated proteins that were electrophoresed, blotted, and tagged with anti-Ro60 or anti-La; positive Ro60 or La bands were marked with a pencil, and then membranes were stripped and reprobed using a Restore™ Western blot stripping buffer (Thermo Fisher Scientific, c. 2109), after washing with Tris-buffered saline with 0.05% Tween 20. The complete removal of primary antibody was demonstrated by lack of chemiluminescent signal in the imager; membranes were blocked with 3% nonfat milk and reprobed with commercial anti-citrulline antibodies, and the possible positive signal at the marked level was recorded.

### 2.12. Antigen Capture Enzyme-Linked Immunosorbent Assay (ACELIA)

Total citrullination activity was detected in cell extracts by ELISA, coating 96 polystyrene microtiter plates (BD Falcon, Bedford, MA) with 100 ng of soluble protein of tissue extracts/well in a volume of 100 *μ*L PBS; active sites were neutralized for 2 hours with 3% BSA, and then citrullination was detected by an anti-citrulline HRP-labelled antibody at 480 nm.

The ACELIA was performed to detect enzymatic activity as previously reported with some modifications [28]. Microtiter 96 polystyrene plates (BD Falcon, Bedford, MA) were coated with anti-PAD2 or PAD4 antibodies at a concentration of 100 ng/well in PBS in a volume of 100 *μ*L. Antibodies were incubated overnight at room temperature, and exposed active sites were neutralized for 2 hours with 3% BSA. The wells were washed with PBS and further incubated for 1 hour at room temperature with 100 *μ*L of the salivary gland extract supernatant containing the PAD enzymes. After incubation, the wells were rinsed repeatedly with washing buffer, and the PAD activity of the captured enzymes was assessed using fibrinogen as a reporter protein. For this assay, 100 ng of fibrinogen (F3879 Sigma, St. Louis, MO) was dissolved in PBS and incubated for 1 hour at 37°C with immunoadsorbent PAD2 and PAD4 enzymes. After incubation, the supernatant from each well was collected, fixed to the wells of new microplates, and incubated overnight at room temperature. The active sites were then blocked with 3% BSA. After washing with PBS, the presence of citrullinated fibrinogen was assessed by ELISA using an anti-citrulline HRP-labelled antibody.

### 2.13. Apoptotic Features

Apoptosis was defined based on two criteria: the classic TUNEL and Annexin assays.

#### 2.13.1. TUNEL

This assay was performed according to the manufacturer's instructions (11684795910, Roche Molecular Biochemicals, Penzberg, Germany). Nuclear stripping of the salivary gland biopsies was performed by immersing the slides for five minutes in 10 mM Tris-HCl, pH 8.0, followed by 15 minutes in 20 *μ*g/mL proteinase K dissolved in 10 mM Tris-HCl. The biopsies were then washed with PBS. The DNA fragments were elongated by incubation for 60 minutes at 37°C with 50 *μ*L of the reaction mixture (DDW, 10x TdT buffer [30 mM Tris base, 140 mM sodium cacodylate, pH 7.2, 1 mM cobalt chloride, and 1 mM DTT], 10% of the final volume), fluorescein-11-dUTP (0.5 mg dissolved in 1 mL of 10 mM Tris-HCl, pH 7.0), and TdT enzyme (0.3 enzyme units/*μ*L). The reaction was terminated with stop solution (300 mM NaCl, 30 mM sodium citrate, pH 8.0), and the slides were examined by fluorescence microscopy.

#### 2.13.2. Annexin Assay

Apoptotic membranes were stained green with FITC Annexin V (BD Pharmingen). This rationale for this assay is based on the loss of cell membrane asymmetry during the early phases of apoptosis. Annexin V is a calcium-dependent phospholipid-binding protein that binds with high affinity to phosphatidylserine. The tissues were rinsed once with a binding buffer, incubated for 15 minutes with 10 *μ*g of FITC-labelled recombinant Annexin, and washed with PBS.

### 2.14. Detection of Ro60 and La Ribonucleoproteins on Apoptotic Membranes Using the Double Fluorescence Assay

Ro and La antigens on apoptotic membranes were detected using double fluorescence assays. Briefly, the Ro60 and La ribonucleoproteins were tagged in red via a 120-minute incubation with an anti-Ro60 or anti-La antibody. The samples were then incubated for 120 minutes with goat anti-mouse IgG Texas red-labelled antibody (sc-2781, Santa Cruz Biotechnology). After washing with PBS, the apoptotic membranes were stained green with FITC Annexin V (BD Pharmingen, USA) as previously described. Finally, the slides were counterstained with DAPI, mounted, and evaluated under a fluorescence microscope using the appropriate filters. Additionally, double fluorescence assays for Ro and La proteins (stained in red) were performed using the tissues that had been processed for TUNEL (apoptotic cell were stained green). In addition, double fluorescence assays were performed to assess the colocalization of Ro or La ribonucleoproteins (stained in green) with citrullinated proteins (stained in red); degree of colocalization areas was analysed by linear regression testing.

### 2.15. Statistical Analysis

The data were processed using the following parametric statistics ANOVA *F*-test and *t*-test, and also linear regression assays were carried with Prism software (GraphPad software). *p* < 0.05 was considered statistically significant.

## 3. Results

### 3.1. Salivary Gland Cultures

**Figure 1:**
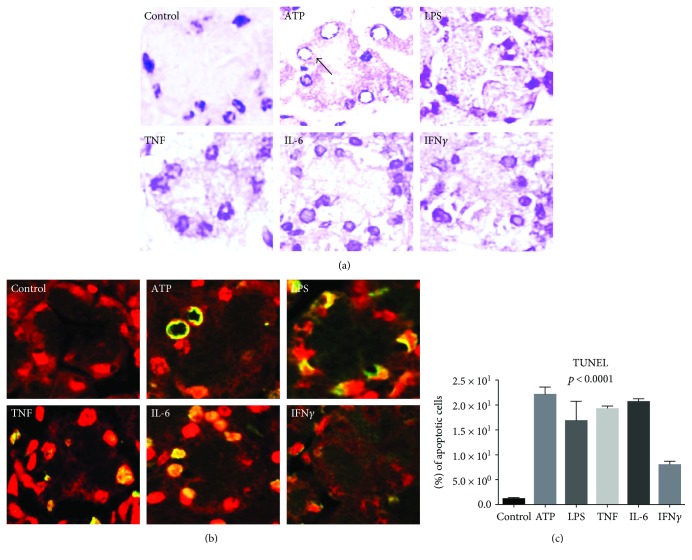
Molecular stimulants induce changes in salivary glands. (a) Morphology of H&E-stained salivary glands from *Balb/c* mice. (b) TUNEL assay (lower panel) showing apoptotic changes in green or yellow in response to different triggers. Nonapoptotic cells were counterstained red with propidium iodide. (c) The graph shows the percentage of apoptotic cells (*p* value calculated by ANOVA).

Excised parotid glands were cultured for 3 hours with excellent viability; however, after stimulation with different molecules, the rate of apoptosis increased from 7% to 22%, in contrast to the controls cultured without additional stimulants with a negligible rate of apoptosis. Remarkably, in response to ATP stimulation, the chromatin adopted a rim-like distribution around the circumference of some nuclei (Figure 1).

### 3.2. PAD2 and PAD4 Proteins Are Induced by Molecular Stimuli

**Figure 2:**
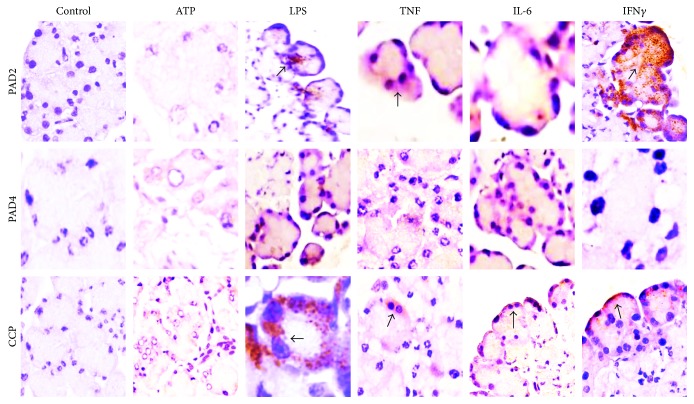
PAD induction by stimulants. PAD2 detection by immunohistochemistry (brown stain). The expression of the enzyme is induced by the stimuli with different molecular triggers.

Under basal conditions, control tissues without any treatment were negative for PAD. However, following stimulation, PAD2 and PAD4 were expressed to a great extent along acini and ductal cells, and both enzymes were expressed similarly in response to different stimuli, excluding LPS and ATP, which induced discrete PAD expression. Nevertheless, a significant difference was detected between controls and stimulated salivary glands (*p* < 0.001; Figure 2).

### 3.3. Relative PAD2, PAD4, Ro60, and La Gene Expression

**Figure 3:**
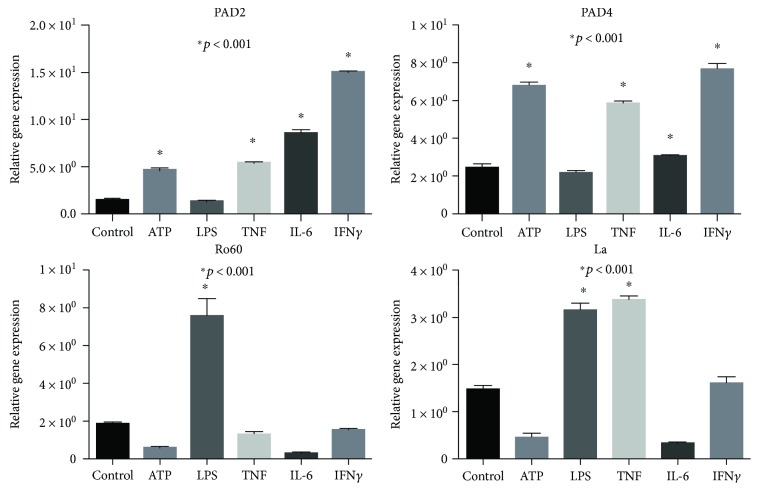
Differential gene expression of PAD2, PAD4, Ro60, and La induced by molecular stimulants ATP, LPS, TNF, IL-6, and IFN*γ* by quantitative PCR (qPCR). Graphs show the validation done by the comparative method (ΔCt). The expression level of each gene was normalized using 18S gen as invariant endogenous control. Asterisk on the top of the bars means that induced gene expression was significantly different with a *p* value of <0.0001 Student *t*-test.

Considering that PAD2 and PAD4 were induced and that they appear to accumulate in response to stimulation, we wondered whether the stimulus was capable of enhancing the transcriptional rates of PADs. We decided to analyze the quantitative gene expression using qPCR, and 18sRNA was selected for validation of genes data. In addition of PAD2 and PAD4 genes, Ro60 and La ribonucleoprotein genes were included in this experiment. As expected, PAD2 showed up of twofold increases in response to all stimulants, while PAD4 augmented significantly with ATP, TNF, and IFN*γ* (*p* < 0.0001). On the other hand, Ro60 expressed threefolds in response to LPS, whereas La ribonucleoprotein amplified under LPS and TNF stimulation (*p* < 0.0001; Figure 3).

### 3.4. Induction of PAD2 and PAD4 Proteins

**Figure 4:**
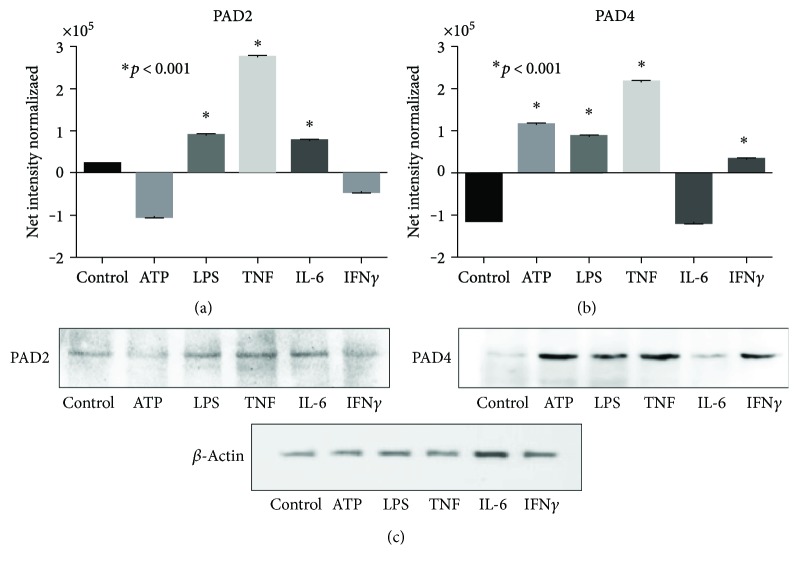
Different molecular triggers induce PAD enzyme expression, as assessed by Western blot analysis. (a) PAD2 enzyme and (b) PAD4 enzyme graphs showing relatively lower levels of PAD2 and PAD4 enzymes induced by molecular triggers. Graphs were normalized by *β*-actin housekeeping protein. (c) Western blot of PAD2, PAD4, and *β*-actin.

To evaluate the expression of PADs in each experimental condition, the protein level of samples was adjusted before loading the electrophoresis gels; thus, the amount of protein was similar for both controls and problems, and then the Western blot bands were analyzed and their values are expressed in pixels—these values were normalized by subtracting the *β*-actin values. As expected, the presence of PAD2 and PAD4 in basal conditions was discrete; however, PAD expression was increased by the effect of certain stressors, for example, PAD2 was increased by TNF stimulation and PAD4 under the effect of ATP, LPS, and TNF (Figure 4).

Regarding the discrepant finding of greater expression of messengers comparing with its cognate protein does not mean that under certain experimental condition, the mRNA level must necessarily predict its protein level: this discrepancy can be explained by mRNA decay and/or protein degradation.

### 3.5. PAD Activity

**Figure 5:**
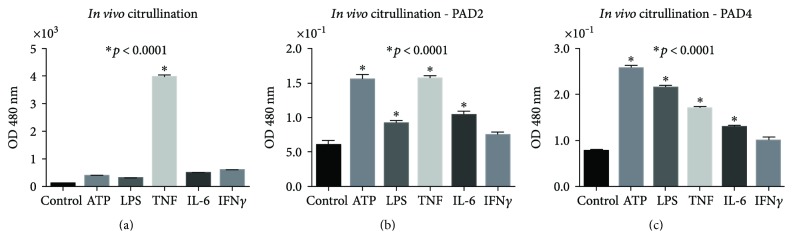
PAD activity. (a) *In vivo* PAD activity measured based on the citrullination of proteins in cell extracts using a direct ELISA assay with tissue extracts fixed onto polystyrene plates and measuring CCP/HRP antibody, this assay shows a significant increase by TNF (*p* < 0.0001). *In vitro* PAD2 activity under basal conditions in response to stimulation with different molecular stimulants; PADs were captured using anti-PAD2 or anti-PAD4 enzymes, then citrullination was detected using an exogenous reporter protein (fibrinogen) and measured with an anti-citrulline-HRP antibody by ACELIA and difference between stimulants and controls (b, c). (b) Significant differences of *in vitro* citrullination PAD2 dependent by the effect of all stimulants except IFN*γ* (*p* < 0.0001); similar results were obtained by PAD4, and bars marked with an asterisk compare control with a specific molecular stimulant by Student *t*-test (c).

We found that citrullinated proteins were widely distributed along the acini and that the ductal epithelium was accessible (Figure 2). Possible PAD activity was explored using two assays: first, using immunohistochemistry, we detected citrullinated proteins at the glandular level *in vivo* using a specific CCP affinity-purified antibody by a direct ELISA that showed a significant citrullination process in tissue extracts stimulated with TNF (*p* < 0.0001; Figure 5(a)). Second, the assessment of the functionality of PAD enzymes *in vitro* with ACELIA revealed significant citrullination of the reporter protein fibrinogen in response to ATP and TNF for PAD2 and ATP, LPS, and TNF for PAD2 and PAD4 (*p* < 0.0001). In contrast, spontaneous citrullination in the absence of a stimulus was negligible (Figures 5(b) and 5(c)).

### 3.6. Induction of Ro60 and La Ribonucleoproteins by Molecular Stimulators

**Figure 6:**
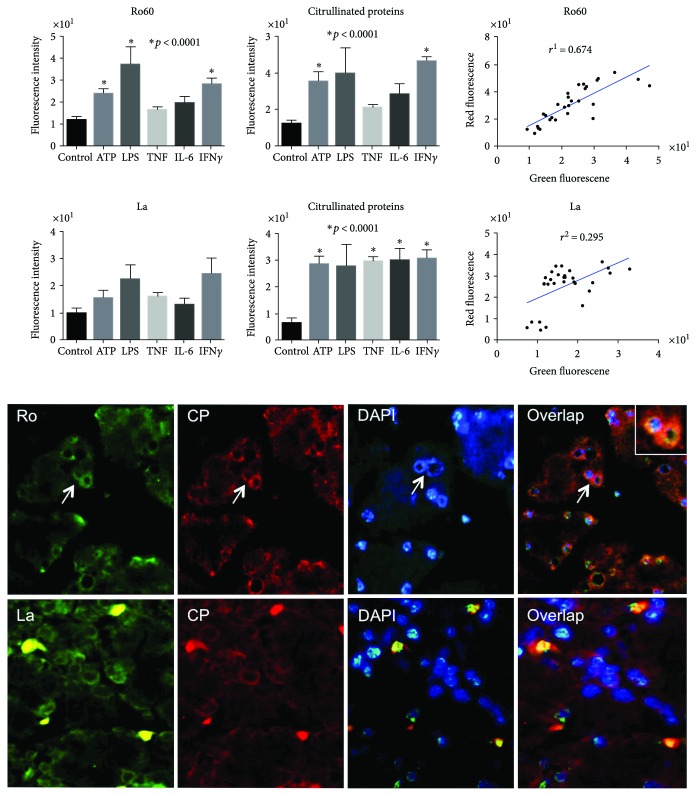
Double fluorescence assays showing colocalization. Graphs show the fluorescent expression of Ro60 and La ribonucleoproteins in response to different molecular triggers. A representative double fluorescence assay of Ro and La (green) colocalization with citrullinated proteins (red). Nuclei are counterstained with DAPI (blue). The green and red fluorescence was analyzed in specific areas of colocation indicated by arrows and magnified in boxes, and the colocalization was analyzed by a regression analysis, which resulted with *r*^2^ of 0.674 for Ro60 meanwile 0.295 for La ribonucleoprotein. The asterisk on the top bars means significant differences from control (*p* < 0.0001, Student *t*-test).

To address whether Ro60 and La ribonucleoproteins were induced *in vitro*, different assays were conducted, and the following main results were obtained. First, Ro60 and La were induced slightly in salivary glands in response to molecular stimuli such as ATP and LPS, as demonstrated by immunofluorescence (Figure 6).

**Figure 7:**
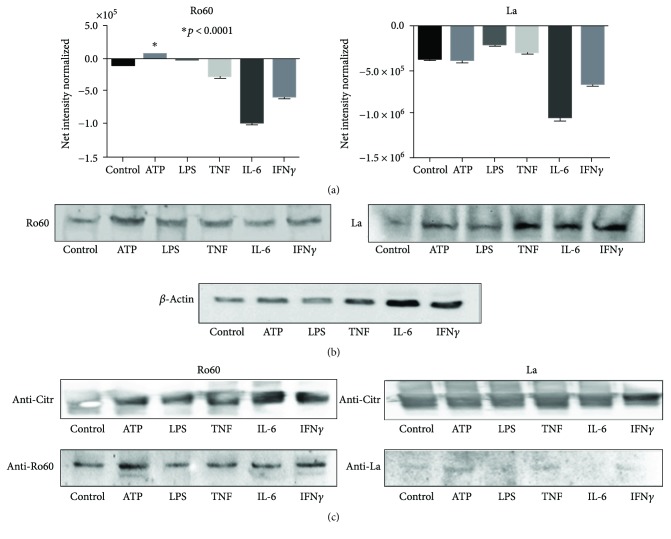
Expression and citrullination of Ro60 and La ribonucleoproteins under stimulation, by Western blot analysis. (a) Graphs show net intensity normalized for the corresponding Ro60, and La bands show a moderate increase of Ro60 induced by ATP (*p* < 0.0001 by *t*-test). La ribonucleoprotein also shows discrete changes regarding its own control without stimulants. (b) Western blots of Ro60, La ribonucleoproteins, and *β*-actin (control protein). (c) Superior panel shows immunoprecipitation assays of citrullinated proteins blotted and probed with anti-Ro or anti-citrulline. Positive bands at 60 kDa (left) and 48 kDa (right) were marked, and membranes were then stripped and reprobed with an anti-Ro60 (left inferior panel) and anti-La (right inferior panel). It is evident that Ro60 had a wide citrullination process specially by ATP and IFN*γ* stimulation. Immunoprecipitates of La ribonucleoprotein display faint citrullination signal under ATP stimulation.

Second, the transcriptional rate of both ribonucleoproteins was apparently induced by molecular stimulants, as seen in Figure 3 by qPCR assay; such increase was true in Ro60 induced by LPS stimuli and La mRNA under LPS and TNF stimulation (*p* < 0.0001; Figure 4). Ro60 and La ribonucleoproteins are widely distributed in all tissues because both are essential for cellular function; however, we must remember that its relative cellular content is small (Figure 7), and if their values are normalized using *β*-actin, in which its cellular content is superior to those of Ro and La, the graphs in consequence will appear as negative values; however, if the nonnormalized values of both ribonucleoproteins are compared with their own basal condition, some molecular stressors such as ATP and LPS may increase their expression as depicted in the Western blots of Figure 7(b).

Another important issue was to demonstrate that Ro and La ribonucleoproteins were citrullinated, and present results demonstrated citrullinated proteins eluted from the immunoadsorbent of Sepharose-4B coupled to anti-citrulline antibodies; therefore, the complex of citrullinated proteins was in the range of 60–48 kDa, and this means that PAD enzymes drive a wide citrullination process including other than Ro60 and La, also probably the albumin can be citrullinated, which is a frequent contaminant of cell extracts and is within the range of molecular weight. However, despite these considerations, our results are specific because after a stringent stripping washings, the positive membranes were marked and reprobed with an anti-Ro or anti-La antibody and this produced a unique and specific band corresponding Ro60 or La (48 kDa); we must mention that some blots of Ro60 showed an additional band below to 60 kDa level, specially those cell extracts from cultures stimulated with ATP or IFN*γ*, and these faint bands may correspond to degradation products of Ro60.

We further tested the specificity of our findings performing an additional immunoprecipitation with Sepharose 4B linked to anti-Ro60 (data not included); then, the immunoprecipitated proteins were probed with an anti-citrulline antibody producing a citrullinated protein at the level of 60 kDa, and this confirmed the first immunoprecipitation assay depicted in Figure 7. In the case of La ribonucleoprotein, we did not obtain the expected results; a possible explanation of the negative results could be due to a lower citrullination rate of La ribonucleoprotein.

Molecular studies demonstrated that Ro60 and La are present in salivary glands under basal conditions, and their relative gene expression by qPCR amplified Ro60 and La mRNAs under certain stimulants such as LPS and TNF; nevertheless, the relative protein content do not increase after stimulation, and Ro60 and La mRNA level did not predict its correspondent protein level.

### 3.7. Ro60 and La Citrullinated Ribonucleoproteins Expressed on Apoptotic Membranes

To address whether molecular stimulants induced citrullination of Ro60 and La, different assays were carried out: first, the effect of PAD activity on Ro60 and La ribonucleoproteins was suggested in the double fluorescent assays, which revealed a high degree of colocalization between the native and citrullinated proteins (r = 0.674), this suggests a broad citrullination process (Figure 6). Second, by immunoprecipitation assay, using an anti-citrulline antibody adsorbed by Sepharose 4B-SPA demonstrated the presence of Ro60 and La ribonucleoproteins; therefore, citrullinated Ro6o ribonucleoprotein was detected even in control extracts (Figure 7(c)). The aforementioned suggests that citrullination of Ro60 ribonucleoprotein occurs *in vivo* at low rate even in control tissues; however, such process is induced under ATP or IFN*γ* stimulation, thus the traces of citrullination of Ro60 ribonucleoprotein in the absence of cellular stressors suggest that this process could occur under physiological condition and we do not know its meaning. Nevertheless, this mechanism may be relevant in autoimmunity only if the rate of citrullination is high as consequence of cell stress that together with an appropriate class II molecule to handle the Ro60 as autoantigen could result in an autoreactive response.

In contrast to La ribonucleoprotein that was immunoprecipitated in cell extracts stimulated with IFN, the citrullination tag was irrelevant and it was faintly recognized by anti-citrulline antibodies in cell extracts stimulated by ATP, suggesting variability in the citrullination of both ribonucleoproteins.

**Figure 8:**
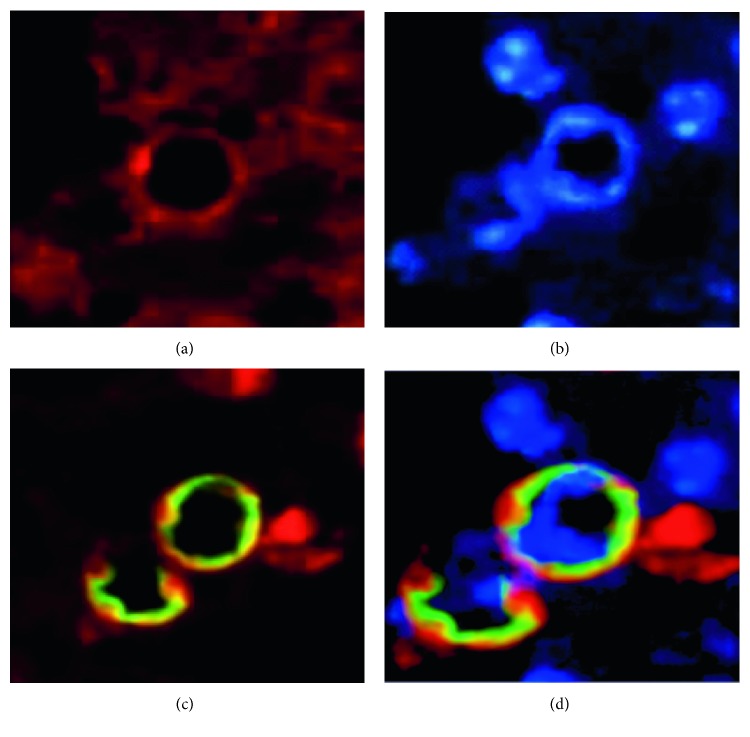
Ro60 expressed in apoptotic cells. (a) Ro60 is stained as red immunofluorescence. (b) Nuclei counterstained with DAPI as blue fluorescence. (c) Double fluorescence Ro ribonucleoprotein (red) in apoptotic cells (green) tagged by TUNEL assay in double fluorescence test. (d) The results of a triple fluorescence show Ro in red outside of apoptotic nuclei in green, and some of the remaining nuclei are stained in blue by DAPI.

**Figure 9:**
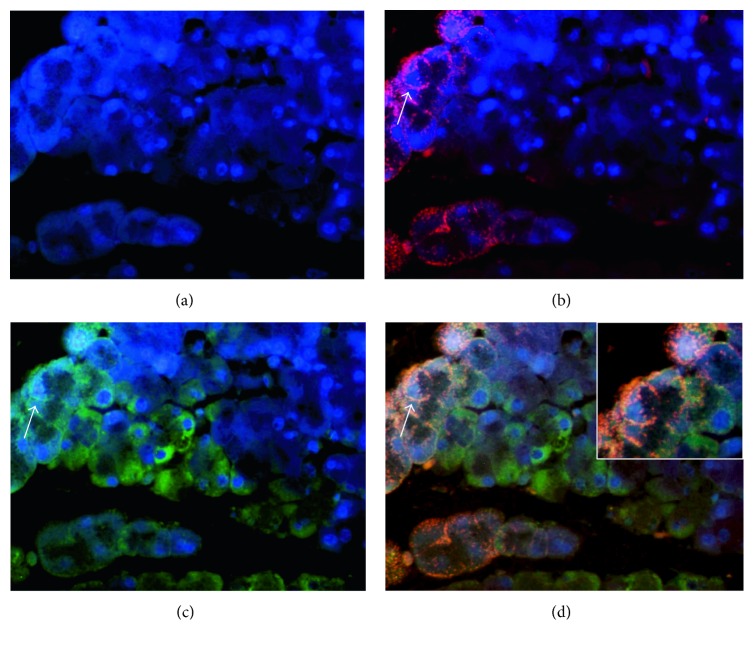
The translocation of Ro60 ribonucleoproteins onto apoptotic membranes as shown in the Annexin assay. (a) Nuclear staining in blue by DAPI. (b) Ro60 stained in red. (c) Apoptotic membranes (green). (d) Arrows correspond to the overlapping image of Annexin (green) with Ro60 (red) in orange (enlarged image in square).

Third, to demonstrate whether Ro60 and La ribonucleoproteins were expressed on the apoptotic membranes of acini and ductal cells, a double fluorescence with TUNEL or Annexin was made (principal results of this assay in Figures 8 and 9).

## 4. Discussion

The present investigation was performed to construct an experimental model to induce the expression of PAD2 and PAD4 enzymes in salivary glands using different triggers. Additionally, the ability of the driven PAD enzymes to induce the posttranslational modification of Ro60 and La ribonucleoproteins was assessed. The main findings suggest that PAD activity may be induced in salivary glands in tissue culture by different triggers and that the induced PAD enzymes are capable of stereochemically modifying the arginine residues of Ro60 and La ribonucleoproteins. Thus, these citrullinated Ro and La ribonucleoproteins may function as autoantigens.

Peptidylarginine deiminase belongs to a family of enzymes that convert the arginine residues of proteins into citrulline. These enzymes are expressed in most mammalian tissues. The PAD2 enzyme isoform is broadly distributed in murine tissues and has been reported to be responsible for most peptidylarginine deiminase activity, which is abrogated in PAD2 knockout mice [22, 29]. Protein citrullination by PAD enzymes can be induced under inflammatory conditions or it can be triggered *in vitro* by LPS, TNF, ATP, or other molecular stimulants [30–33]. Nevertheless, this induction depends on the PAD substrate because the specificities of PAD2 and PAD4 are distinct [34, 35]. The present study demonstrated that PAD2 and PAD4 are broadly expressed in the salivary gland of *Balb/c* mice. However, PAD4 was the dominant enzyme, and the interaction between PAD4 with protein tyrosine phosphatase enhances protein citrullination [36, 37].

Regarding the presence of Ro and La ribonucleoproteins in murine salivary glands, both ribonucleoproteins were widely distributed in acinar and ductal cells, because they normally perform a function in the cellular physiology. Also, we demonstrated that Ro60 and La ribonucleoproteins are citrullinated in this model; however, their hypercitrullination can be of pathogenic importance because this process may foster the production of autoantigens.

The potential effect of Ro60 citrullination has been tested *in vitro* by other investigators using synthetic peptides, the arginine residues 174 and 184 were transformed into citrulline, and the main effect of this modification was an increase in the antibody binding strength in comparison to unmodified linear epitopes [38]. Besides, the results from a proteomic analysis demonstrated that a broad repertoire of proteins, including La ribonucleoprotein, can be posttranslationally modified in the salivary glands of patients with Sjögren's syndrome [39]. The aforementioned observations are consistent with the present findings.

We have previously noted that posttranslational protein modifications that are dependent on PAD may occur in the salivary glands of patients with Sjögren's syndrome, and this mechanism could be important for disease pathogenesis because salivary glands constitute an accessible target via the salivary ducts for different environmental triggers. Consequently, external stimulation can activate PAD enzymes, which in turn can modify proteins that may contribute to the autoimmune response. We believe that citrullination is a formidable catalytic mechanism that underlies the production of autoantigens that can potentially abrogate immune tolerance.

Considering the difficulty associated with obtaining patient salivary glands to assess potential inducers of PAD activity, we designed the present experimental approach using salivary glands of *Balb/c* mice to answer our primary question. Our results suggest that the bacterial products of Gram-negative bacteria such as LPS are capable of inducing PAD activity. Similarly, some proinflammatory cytokines, such as TNF produced by local inflammation, may activate PAD enzymes and citrullinate Ro and La ribonucleoproteins *in situ*. Therefore, the reason for using a serum of a primary Sjögren's syndrome with CCP antibody and positive for anti-Ro60 and anti-La antibodies, as well as the lack of commercial antibodies against citrullinated nonhistone proteins, encouraged us to purify CCP antibodies to identify possible areas of citrulination in parotids of *Balb/c* mice, since the human CCP antibodies have been successfully used by others for tissue labelling [40]. Consequently, our proposal rather than an animal model of Sjögren's disease is a tool to demonstrate how the enzymatic PAD machinery can posttranslationally modify normal proteins by transforming them into possible autoantigens as a way to understand one of the multiple triggers of autoimmunity.

In addition, molecular triggers such as ATP, LPS, and TNF induce apoptosis in acini and ducts, and under certain experimental conditions, ATP and LPS trigger the inflammasome pathway, resulting in apoptosis [14, 41, 42]. In the present study, different degrees of apoptosis were induced in salivary glands. This finding is not surprising; however, it is important to mention that Ro60 and La autoantigens were translocated to the cell surface on apoptotic membranes, which suggests that, via apoptosis, Ro and La intracellular autoantigens become accessible to the antigen-presenting cells.

## 5. Conclusions

Based on our findings, it is possible to hypothesize that an initial and nonspecific external trigger (microorganisms, chemical products, or another type of trigger) can activate the PAD pathway, changing normal proteins into possible autoantigens. Citrullinated proteins in primed autoimmune class II MHC molecules and autoreactive lymphocyte clones can generate an autoimmune response similar to that of Sjögren's syndrome. However, this hypothesis must be further explored.

## Abbreviations

ACELIA: Antigen capture enzyme-linked immunosorbent assay
CCP antibodies: Anti-cyclic citrullinated peptide
CCPs: Synthetic cyclic citrullinated peptides
DMEM: Dulbecco's modified Eagle's medium
FBS: Foetal bovine serum
HRP: Horseradish peroxidase
IFN: Interferon
PAD: Peptidylarginine deiminase enzymes
PBS: Phosphate-buffered saline
PMSF: Phenylmethylsulfonyl fluoride
pNPP: P-nitro phenyl phosphate, disodium salt
RA: Rheumatoid arthritis
RT-PCR: Reverse-transcription/polymerase chain reaction.

## References

[1] Eggleton P., Haigh R., Winyard P. G. (2008). Consequence of neo-antigenicity of the ‘altered self’. *Rheumatology*.

[2] Guerrin M., Ishigami A., Méchin M. (2003). cDNA cloning, gene organization and expression analysis of human peptidylarginine deiminase type I. *Biochemical Journal*.

[3] Litt M., Qiu Y., Huang S. (2009). Histone arginine methylations: their roles in chromatin dynamics and transcriptional regulation. *Bioscience Reports*.

[4] Morgan H. D., Santos F., Green K., Dean W., Reik W. (2005). Epigenetic reprogramming in mammals. *Human Molecular Genetics*.

[5] Arita K., Shimizu T., Hashimoto H., Hidaka Y., Yamada M., Sato M. (2006). Structural basis for histone N-terminal recognition by human peptidylarginine deiminase 4. *Proceedings of the National Academy of Sciences of the United States of America*.

[6] Coudane F., Mechin M. C., Huchenq A. (2011). Deimination and expression of peptidylarginine deiminases during cutaneous wound healing in mice. *European Journal of Dermatology*.

[7] Wang S., Wang Y. (2013). Peptidylarginine deiminases in citrullination, gene regulation, health and pathogenesis. *Biochimica et Biophysica Acta (BBA)-Gene Regulatory Mechanisms*.

[8] Nocturne G., Mariette X. (2013). Advances in understanding the pathogenesis of primary Sjögren’s syndrome. *Nature Reviews Rheumatology*.

[9] Gottenberg J., Mignot S., Nicaise-Rolland P. (2005). Prevalence of anti-cyclic citrullinated peptide and anti-keratin antibodies in patients with primary Sjögren’s syndrome. *Annals of the Rheumatic Diseases*.

[10] Herrera-Esparza R., Rodríguez-Rodríguez M., Pérez-Pérez M. E. (2013). Posttranslational protein modification in the salivary glands of Sjögren’s syndrome patients. *Autoimmune Diseases*.

[11] Chen X., Taylor D. W., Galan J. E., Wang H. W., Wolin S. L. (2013). An RNA degradation machine sculpted by Ro autoantigen and noncoding RNA. *Cell*.

[12] Liang C., Xiong K., Szulwach K. E. (2013). Sjögren syndrome antigen B (SSB)/La promotes global microRNA expression by binding microRNA precursors through stem-loop recognition. *The Journal of Biological Chemistry*.

[13] Henriksson G. (2014). Presymptomatic autoantibodies in Sjögren’s syndrome: what significance do they hold for the clinic?. *Expert Review of Clinical Immunology*.

[14] Miglio G., Veglia E., Fantozzi R. (2015). Fumaric acid esters prevent the NLRP3 inflammasome-mediated and ATP-triggered pyroptosis of differentiated THP-1 cells. *International Immunopharmacology*.

[15] Hecker A., Küllmar M., Wilker S. (2015). Phosphocholine-modified macromolecules and canonical nicotinic agonists inhibit ATP-induced IL-1*β* release. *The Journal of Immunology*.

[16] Cunningham C. C., Corr E. M., Cox D. J., Dunne A. (2015). Investigating inflammasome activation under conditions of cellular stress and injury. *Methods in Molecular Biology*.

[17] Bizzaro N., Tonutti E., Tozzoli R., Villalta D. (2007). Analytical and diagnostic characteristics of 11 2nd- and 3rd-generation immunoenzymatic methods for the detection of antibodies to citrullinated proteins. *Clinical Chemistry*.

[18] Esparza R. H., Swaak T., Aarden L., Smeenk R. (1985). Complement-fixing antibodies to dsDNA detected by the immunofluorescence technique on *Crithidia luciliae*. A critical appraisal. *Journal of Rheumatology*.

[19] Bradford M. M. (1976). A rapid and sensitive method for the quantitation of microgram quantities of protein utilizing the principle of protein-dye binding. *Analytical Biochemistry*.

[20] Avrameas S., Ternynck T. (1971). Peroxidase labelled antibody and Fab conjugates with enhanced intracellular penetration. *Immunochemistry*.

[21] Bollain-Y-Goytia J. J., Mendoza‐Salazar L., Romo‐Flores M. (2008). The presence of citrulline in salivary glands is evidence that nitric oxide is mediator of inflammation in Sjögren acinar epithelia. *International Journal of Rheumatic Diseases*.

[22] Arandjelovic S., McKenney K. R., Leming S. S., Mowen K. A. (2012). ATP induces protein arginine deiminase 2-dependent citrullination in mast cells through the P2X7 purinergic receptor. *The Journal of Immunology*.

[23] Spurlock C. F., Tossberg J. T., Guo Y., Sriram S., Crooke P. S., Aune T. M. (2015). Defective structural RNA processing in relapsing-remitting multiple sclerosis. *Genome Biology*.

[24] Vossenaar E. R., Radstake T. R., van der Heijden A. (2004). Expression and activity of citrullinating peptidylarginine deiminase enzymes in monocytes and macrophages. *Annals of the Rheumatic Diseases*.

[25] Jain M., Nijhawan A., Tyagi A. K., Khurana J. P. (2006). Validation of housekeeping genes as internal control for studying gene expression in rice by quantitative real-time PCR. *Biochemical and Biophysical Research Communications*.

[26] Laemmli U. K. (1970). Cleavage of structural proteins during the assembly of the head of bacteriophage T4. *Nature*.

[27] Towbin H., Staehelin T., Gordon J. (1979). Electrophoretic transfer of proteins from polyacrylamide gels to nitrocellulose sheets: procedure and some applications. *Proceedings of the National Academy of Sciences of the United States of America*.

[28] Damgaard D., Senolt L., Nielsen M. F., Pruijn G. J., Nielsen D. H. (2014). Demonstration of extracellular peptidylarginine deiminase (PAD) activity in synovial fluid of patients with rheumatoid arthritis using a novel assay for citrullination of fibrinogen. *Arthritis Research & Therapy*.

[29] van Beers J. J., Zendman A. J., Raijmakers R., Stammen-Vogelzangs J., Pruijn G. J. (2013). Peptidylarginine deiminase expression and activity in PAD2 knock-out and PAD4-low mice. *Biochimie*.

[30] Koziel J., Bryzek D., Sroka A. (2014). Citrullination alters immunomodulatory function of LL-37 essential for prevention of endotoxin-induced sepsis. *The Journal of Immunology*.

[31] Ferrari-Lacraz S., Sebbag M., Chicheportiche R., Foulquier C., Serre G., Dayer J. M. (2012). Contact with stimulated T cells up-regulates expression of peptidylarginine deiminase 2 and 4 by human monocytes. *European Cytokine Network*.

[32] Neeli I., Khan S. N., Radic M. (2008). Histone deimination as a response to inflammatory stimuli in neutrophils. *The Journal of Immunology*.

[33] Lee H. J., Joo M., Abdolrasulnia R. (2010). Peptidylarginine deiminase 2 suppresses inhibitory *κ*B kinase activity in lipopolysaccharide-stimulated RAW 264.7 macrophages. *The Journal of Biological Chemistry*.

[34] Abdullah S., Farmer E., Spargo L., Logan R., Gully N. (2013). Porphyromonas gingivalis peptidylarginine deiminase substrate specificity. *Anaerobe*.

[35] Assohou-Luty C., Raijmakers R., Benckhuijsen W. E. (2014). The human peptidylarginine deiminases type 2 and type 4 have distinct substrate specificities. *Biochimica et Biophysica Acta (BBA)-Proteins and Proteomics*.

[36] Chang H. H., Dwivedi N., Nicholas A. P., Ho I. (2015). The W620 polymorphism in PTPN22 disrupts its interaction with peptidylarginine deiminase type 4 and enhances citrullination and NETosis. *Arthritis & Rheumatology*.

[37] Reyes-Castillo Z., Palafox-Sánchez C. A., Parra-Rojas I. (2015). Comparative analysis of autoantibodies targeting peptidylarginine deiminase type 4, mutated citrullinated vimentin and cyclic citrullinated peptides in rheumatoid arthritis: associations with cytokine profiles, clinical and genetic features. *Clinical & Experimental Immunology*.

[38] Terzoglou A. G., Routsias J. G., Moutsopoulos H. M., Tzioufas A. G. (2006). Post-translational modifications of the major linear epitope 169-190aa of Ro60 kDa autoantigen alter the autoantibody binding. *Clinical & Experimental Immunology*.

[39] Stea E. A., Routsias J. G., Samiotaki M. (2007). Analysis of parotid glands of primary Sjögren’s syndrome patients using proteomic technology reveals altered autoantigen composition and novel antigenic targets. *Clinical & Experimental Immunology*.

[40] Vossenaar E. R., Smeets T. J., Kraan M. C., Raats J. M., van Venrooij W. J., Tak P. P. (2004). The presence of citrullinated proteins is not specific for rheumatoid synovial tissue. *Arthritis & Rheumatology*.

[41] Vajjhala P. R., Lu A., Brown D. L. (2015). The inflammasome adaptor ASC induces procaspase-8 death effector domain filaments. *The Journal of Biological Chemistry*.

[42] Stammler D., Eigenbrod T., Menz S. (2015). Inhibition of histone deacetylases permits lipopolysaccharide-mediated secretion of bioactive IL-1*β* via a caspase-1-independent mechanism. *The Journal of Immunology*.

